# 8-Furyl­imidazolo-2′-de­oxy­cytidine: crystal structure, packing, atropisomerism and fluorescence

**DOI:** 10.1107/S2053229622001000

**Published:** 2022-02-09

**Authors:** Simone Budow-Busse, Sunit K. Jana, Dasharath Kondhare, Constantin Daniliuc, Frank Seela

**Affiliations:** a Laboratory of Bioorganic Chemistry and Chemical Biology, Center for Nanotechnology, Heisenbergstrasse 11, 48149 Münster, Germany; b Organisch-Chemisches Institut, Westfälische Wilhelms-Universität Münster, Corrensstrasse 40, 48149 Münster, Germany; c Laboratorium für Organische und Bioorganische Chemie, Institut für Chemie, Universität Osnabrück, Barbarastrasse 7, 49069 Osnabrück, Germany

**Keywords:** 8-furyl­imidazolo-2′-de­oxy­cytidine, crystal structure, atropisomerism, crystal packing, Hirshfeld surface analysis, fluorescence

## Abstract

8-Furyl­imidazolo-2′-de­oxy­cytidine (^fur^ImidC), C_14_H_14_N_4_O_5_, is a fluorescent analogue of 2′-de­oxy­cytidine, also displaying the same recognition face. As a constituent of DNA, ^fur^ImidC forms extraordinarily strong silver-mediated self-pairs. Crystal structure determination revealed that ^fur^ImidC adopts two types of disordered residues: the sugar unit and the furyl moiety. The disorder of the sugar residue amounts to an 87:13 split. The disorder of the furyl ring results from axial chirality at the C8—C2′′ bond connecting the nucleobase to the hetero­cycle. The two atropisomers are present in unequal proportions [occu­pancies of 0.69 (2) and 0.31 (2)], and the nucleobase and the furyl moiety are coplanar. Considering the atomic sites with predominant occupancy, an *anti* conformation with χ = − 147.2 (7)° was found at the glycosylic bond and the 2′-de­oxy­ribosyl moiety shows a C2′-*endo* (*S*, ^2^ *T*_1_) conformation, with *P* = 160.0°. A ^1^H NMR-based conformational analysis of the furan­ose puckering revealed that the *S* conformation predominates also in solution. In the solid state, two neighbouring ^fur^ImidC mol­ecules are arranged in a head-to-tail fashion, but with a notable tilt of the mol­ecules with respect to each other. Consequently, one N—H⋯N hydrogen bond is found for neighbouring mol­ecules within one layer, while a second N—H⋯N hydrogen bond is formed to a mol­ecule of an adjacent layer. In addition, hydrogen bonding is observed between the nucleobase and the sugar residue. A Hirshfeld surface analysis was performed to visualize the inter­molecular inter­actions observed in the X-ray study. In addition, the fluores­cence spectra of ^fur^ImidC were measured in solvents of different polarity and viscosity. ^fur^ImidC responds to microenvironmental changes (polarity and viscosity), which is explained by a hindered rotation of the furyl residue in solvents of high viscosity.

## Abstract 2

A disproportionate disorder is observed for the furyl ring of 8-furyl­imidazolo-2′-de­oxy­cytidine (^fur^ImidC) due to atropisomerism, with the two isomers related to each other by a 180° rotation about the C8—C2′′ bond. ^fur^ImidC shows an *anti* conformation at the glycosyl bond and a C2′-*endo* sugar pucker of the 2′-de­oxy­ribosyl moiety. The mol­ecule is fluorescent and the fluorescence responds to solvent viscosity.

## Introduction

**Figure 1:**
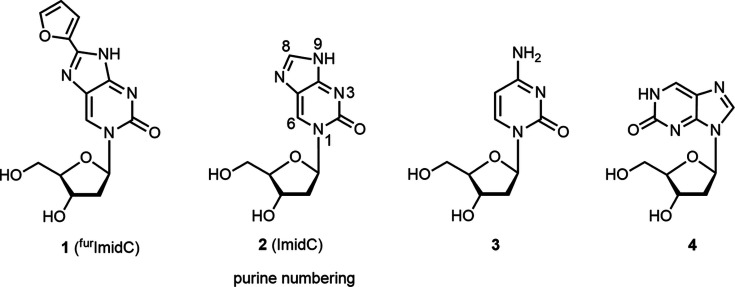
Imidazolo-dC nucleosides and close derivatives.

8-Furyl­imidazolo-2′-de­oxy­cytidine (^fur^ImidC, **1**) is a base-modified nucleoside with the recognition face of 2′-de­oxy­cytidine (dC, **5**) (Jana *et al.*, 2015 ▸) (purine numbering is used throughout this article). This nucleoside displays a strong fluorescence as it is com­posed of the fluorescent nucleobase 2-hy­droxy­purine carrying a furyl system at the 8-position and a sugar moiety linked to the N atom at the 1-position (Fig. 1 ▸). The related nucleoside 2′-de­oxy­isoinosine (**3**), with the sugar moiety linked to the 9-position, is also fluorescent (Seela *et al.*, 1994 ▸, 2000 ▸; Seela & Chen, 1995 ▸). As a constituent of DNA, ^fur^ImidC (**1**) shows unique properties, as it forms strong base pairs with dG and extremely strong silver-mediated self-pairs (Jana *et al.*, 2015 ▸).

Imidazolo-2′-de­oxy­cytidine (**2**) is structurally related to pyrrolo-2′-de­oxy­cytidine (pyrrolo-dC, **4**), a fluorescent dC congener with a pyrrolo­[2,3-*d*]pyrimidine instead of the purine skeleton of **2**. Pyrrolo-dC (**4**) (Inoue *et al.*, 1987 ▸) has been incorporated into DNA and base pairs selectively with dG without disturbing the Watson–Crick duplex structure (Hudson & Ghorbani-Choghamarani, 2007 ▸; Wilhelmsson, 2010 ▸).

While much work has been performed on pyrrolo-dC and its derivatives (Tinsley & Walter, 2006 ▸; Wahba *et al.*, 2010 ▸; Ming & Seela, 2012 ▸; Noé *et al.*, 2012 ▸), reports of studies on ImidC (**2**) are rare. Fischer and co-workers synthesized a series of *para*-substituted imidazolo­cytidines and incorporated 8-(*p*-CF_3_-phen­yl)imidazolo-2′-de­oxy­cytidine into oligonu­cleo­tides to study mismatch discrimination (Kovaliov *et al.*, 2013 ▸, 2014 ▸). Recently, it was shown that substituted imidazolo-2′-de­oxy­cytidines can function as effective silver-ion binders (Mei *et al.*, 2014 ▸). In DNA, self-pairs of imidazolo-2′-de­oxy­cytidines form silver-mediated base pairs in which silver ions take over the function of protons in semiprotonated ‘dC–dC’ base pairs (Mei *et al.*, 2014 ▸; Clever *et al.*, 2007 ▸). In this context, 8-furyl­imidazolo-2′-de­oxy­cytidine (^fur^ImidC, **1**), decorated with a furyl substituent at the 8-position (Fig. 1 ▸), was designed (Jana *et al.*, 2015 ▸). The furyl moiety is anti­cipated to enhance the coordination forces for silver ions to the system. Also, the fluorescence properties of **1** are improved com­pared to the unmodified ImidC (**2**).

In addition, the ^fur^ImidC (**1**) mol­ecule com­prises a distinctive feature, namely, the presence of the furyl substituent, which enables the mol­ecule to form atropisomers. Atropisomerism is a dynamic type of axial chirality with stereochemically hindered rotation about single bonds and can generate a mixture of two isomers.

To verify the formation of atropisomers, an X-ray analysis of 8-furyl­imidazolo-2′-de­oxy­cytidine (**1**) was performed. The conformation of ^fur^ImidC (**1**) in the solid state and in solution, as well as the crystal packing of the mol­ecule, were studied. Solvent-dependent fluorescence spectra of **1** were determined to monitor the impact of microenvironmental changes in solution.

## Experimental

### Synthesis and crystallization of ^fur^ImidC (1)

8-Furyl­imidazolo-dC (**1**) was synthesized as reported by Jana *et al.* (2015 ▸). For crystallization, com­pound **1** was dissolved in water and was obtained as colourless needles by slow evaporation of the solvent at room temperature. A needle-like specimen of **1** was used for the X-ray crystallographic analysis.

### Refinement

Crystal data, data collection and structure refinement details are summarized in Table 1 ▸. The H atom at the N9 atom was refined freely. Moreover, the furyl ring and the sugar moiety linked to atom N1 are disordered over two positions. Several restraints (*SHELXL* instructions SADI, SAME, ISOR and SIMU) were used in order to improve refinement stability. Full details of the refinement instructions can be found embedded in the CIF.

## Results and discussion

### Mol­ecular geometry and conformation of 8-furyl­imidazolo-dC (1)

**Figure 2:**
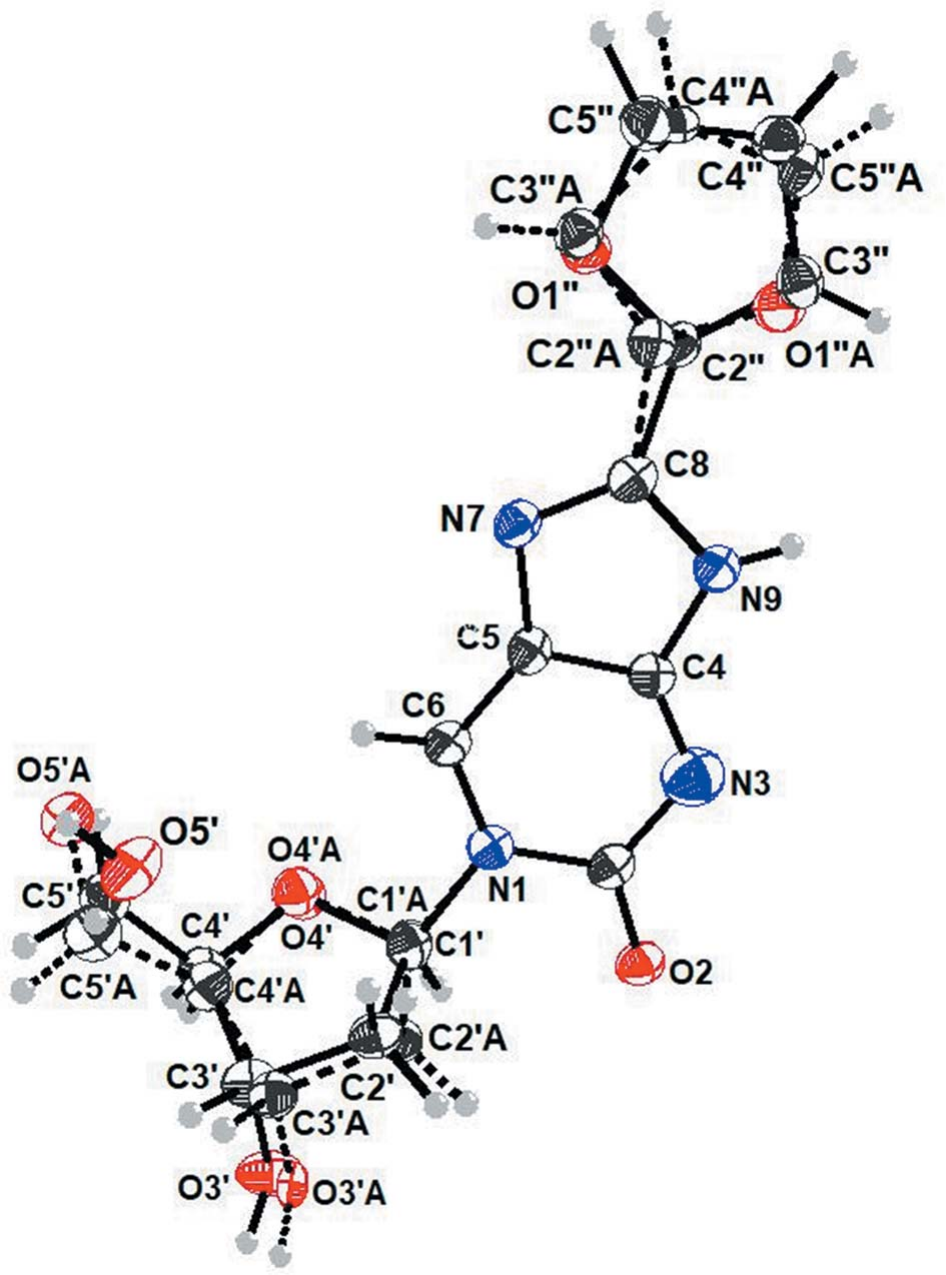
Perspective view of 8-furyl­imidazolo-dC (**1**), showing the atomic num­bering scheme. The major disorder com­ponent has been drawn using full lines and the minor disorder com­ponent has been drawn using dashed lines. Displacement ellipsoids are drawn at the 50% probability level and H atoms are shown as small spheres of arbitrary size.

The crystals of ^fur^ImidC (**1**) are ortho­rhom­bic with the space group *P*2_1_2_1_2_1_. For selected geometric parameters, see Table 2 ▸. The three-dimensional (3D) structure of ^fur^ImidC (**1**) is shown in Fig. 2 ▸ and shows two sets of disordered groups. The furyl group is rotationally disordered over two positions having occupancies of 0.69 (2) (solid line) and 0.31 (2) (dashed line). The second disorder concerns the sugar residue, but it is less pronounced, with occupancies of 0.86 (7) and 0.13 (3).

**Figure 3:**
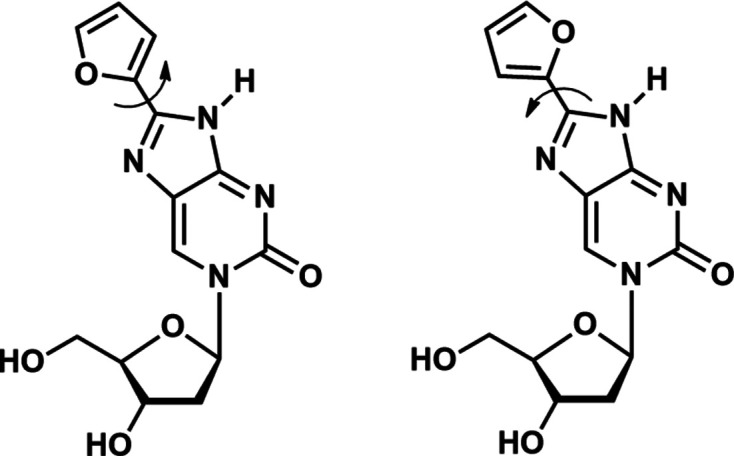
Atropisomerism of 8-furyl­imidazolo-2′-de­oxy­cytidine (**1**).

The disordered sites of the furyl ring are related to each other by a rotation of 180° about the C8—C2′′ bond (Fig. 3 ▸). Accordingly, the two isomers are atropisomers (Clayden *et al.*, 2009 ▸; Toenjes & Gustafson, 2018 ▸) and the C8—C2′′ bond is a chiral axis. Considering the com­position of the entire ^fur^ImidC (**1**) mol­ecule including the attached sugar residue, the isomers are diastereomeric com­prising different physical properties. This might also contribute to the unequal proportion of both atropisomers within the crystal. Most inter­estingly, different to other nucleosidic com­pounds forming atropisomers, *e.g.* pyr­rolo­pyrimidine-based (PPY) kinase inhibitors (Smith *et al.*, 2015 ▸), the nucleobase and the furyl ring of ^fur^ImidC (**1**) are coplanar in both atropisomers. The crystal struc­ture of a PPY inhibitor showed a perpendicular arrange­ment of the nucleobase and the attached benzyl ring (Smith *et al.*, 2015 ▸). This difference can probably be attributed to the fact that the furyl ring of ^fur^ImidC (**1**) does not carry any bulky substituents. This lowers the energy barrier for atropisomer inter­convertion of ^fur^ImidC (**1**) to such an extent that both isomers become inseparable in solution at room tem­per­ature.

As the nucleobase shows no disorder and the sugar residue adopts only a minor disorder, the conformational analysis of ^fur^ImidC (**1**) was carried out mainly considering the atomic sites with predominant occupancy. The torsion angle χ (O4′—C1′—N1—C2) (IUPAC–IUB Joint Commission on Biochemical Nomenclature, 1983 ▸) of ^fur^ImidC (**1**) is defined in analogy to pyrimidine nucleosides, as this mol­ecule can be considered a pyrimidine nucleoside analogue with a fused imidazole ring and a furyl substituent. The orientation of the nucleobase with oxygen-2 of the pyrimidine ring pointing away from the sugar moiety refers to an *anti* conformation (Saenger, 1984 ▸). The *syn* conformation is adopted when oxygen-2 is pointing towards the sugar ring. Natural pyrimidine 2′-de­oxy­ribonucleosides, including 2′-de­oxy­cytidine (**5**), prefer an *anti* conformation (Young & Wilson, 1975 ▸). Also for ^fur^ImidC (**1**), an *anti* conformation is found around the glycosylic bond, with χ = −147.2 (7)°.

Moreover, the 8-furyl substituent of **1** is connected to the imidazole residue *via* an ar­yl–aryl bond and is coplanar with the nucleobase. In the solid state, the C8—C2′′ bond becomes a chiral axis due to atropisomerism. The bond length is 1.451 (11) Å for the major atropisomer and somewhat shorter for the minor atropisomer [C8—C2′′*A* = 1.418 (19) Å] (Table 2 ▸). The length of the glycosylic C1′—N1 bond connecting the nucleobase and sugar is 1.477 (8) Å (Table 2 ▸). This is in the range of the average length (1.49 Å) of glycosylic bonds observed for pyrimidine nucleosides (Saenger, 1984 ▸).

The orientation of the exocyclic 5′-hy­droxy group relative to the sugar ring is characterized by the torsion angle γ (O5′—C5′—C4′—C3′) (Saenger, 1984 ▸). For ^fur^ImidC (**1**), a synclinal (+*sc*) conformation with γ = 47.3 (10)° is observed, which is the preferred conformation in pyrimidine nucleosides (Saenger, 1984 ▸).

The sugar pucker of the 2′-de­oxy­ribofuanosyl moiety is another major parameter for characterizing the conformation of nucleosides. In correlation to the major displacement of C3′ or C2′ from the median plane of C1′—O4′—C4′, nucleosides can adopt two principal sugar puckering modes, namely, C3′-*endo* (*N*) and C2′-*endo* (*S*) (Altona & Sundaralingam, 1972 ▸; Saenger, 1984 ▸). The C2′-*endo* conformation is the preferred puckering mode of canonical 2′-de­oxy­ribonucleosides. Also, the sugar moiety of ^fur^ImidC (**1**) adopts C2′-*endo* (*S*, ^2^ *T*_1_) conformation with a pseudorotational phase angle *P* = 160.0° and a maximum amplitude τ_m_ = 35.6° (Altona & Sundaralingam, 1972 ▸; Saenger, 1984 ▸).

However, the solid-state conformation of nucleosides is not necessarily identical to the nucleoside conformation observed in solution or as constituents of DNA. The population of *S versus N* conformers is rapidly inter­converting in solution and shows a preference for one conformation (Saenger, 1984 ▸). In this regard and to confirm the sugar conformation of ^fur^ImidC (**1**) in solution, a conformational analysis of the furan­ose puckering of ^fur^ImidC (**1**) was performed. For this, a high-resolution ^1^H NMR spectrum of com­pound **1** was measured in dimethyl sulfoxide (DMSO) and coupling constants were determined. The ^1^H NMR spectrum (Fig. S4) and the coupling constants (Table S2) are available in the supporting information. The program *PSEUROT* (Version 6.3; Van Wijk *et al.*, 1999 ▸) was used to carry out the conformational analysis of the sugar puckering of the 2′-de­oxy­ribo­furanosyl ring. This pro­gram calculates the population of the *N*- and *S*-type conformers on the basis of five ^3^ *J*(H,H) coupling constants, namely, ^3^ *J*(H1′,H2′), ^3^ *J*(H1′,H2"), ^3^ *J*(H2′,H3′), ^3^ *J*(H2′′,H3′) and ^3^ *J*(H3′,H4′). The *PSEUROT* analysis of ^fur^ImidC (**1**) showed that the 2′-de­oxy­ribo­furanosyl moiety of this nucleoside prefers the *S* conformation (57%) in solution. However, com­pared to the canonical 2′-de­oxy­cytidine (**5**) (72% *S*), the *S* population of **1** is less pronounced (Budow-Busse *et al.*, 2021 ▸).

Taken together, despite the fact that com­pound **1** carries a nucleobase consisting of a pyrimidine moiety with a fused imidazole ring (corresponding to N1-glycosyl­ated 2-hy­droxy­purine), this nucleoside adopts conformational properties typical for pyrimidine 2′-de­oxy­ribonucleosides.

### Hydrogen bonding and mol­ecular packing of ^fur^ImidC (1)

**Figure 4:**
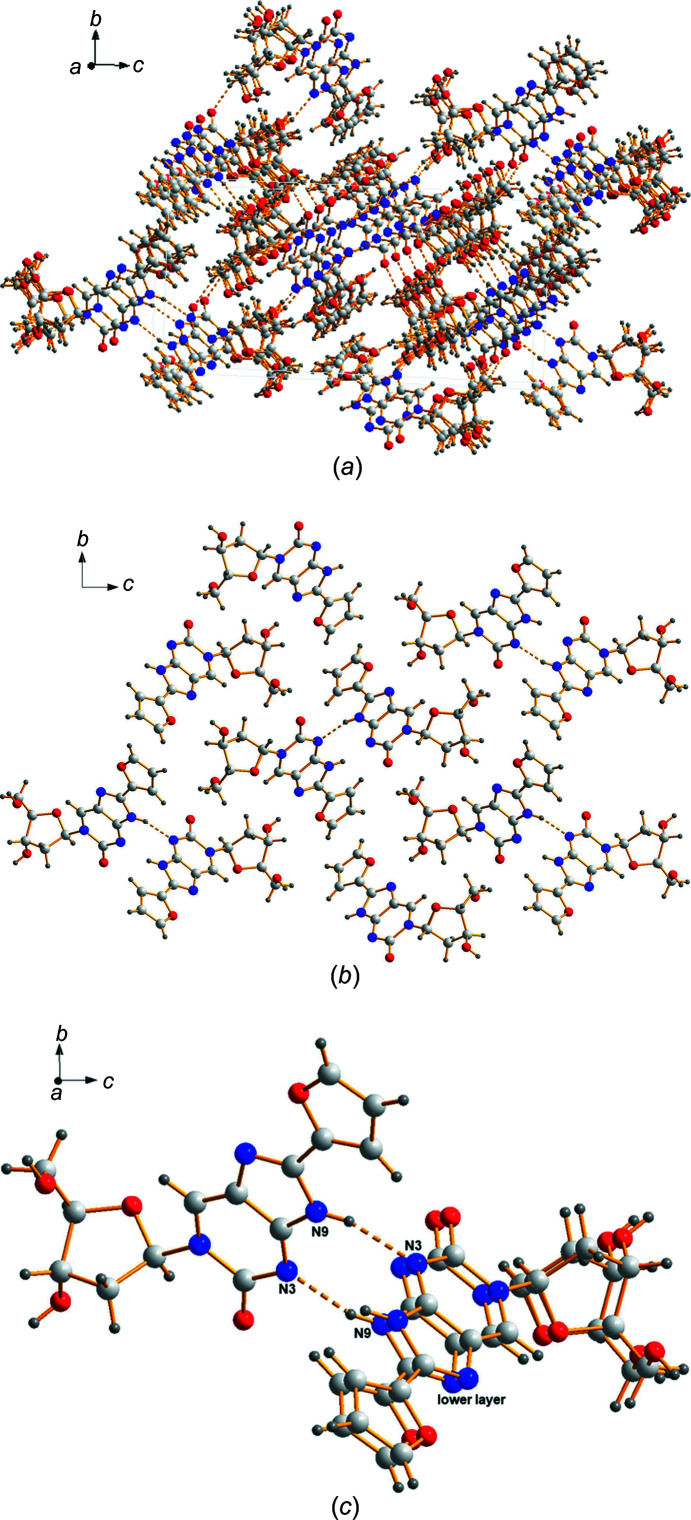
(*a*) Multilayered packing of **1**, showing the stacking inter­actions of the mol­ecules. (*b*) Hydrogen bonding of ^fur^ImidC (**1**) within the *bc* plane. (*c*) Self-pairing of ^fur^ImidC (**1**), with a head-to-tail alignment, and showing the contacts of one mol­ecule to two neighbouring mol­ecules. For clarity, the minor disorder com­ponents have been omitted from parts (*b*) and (*c*).

Within the extended network, the mol­ecules of ^fur^ImidC (**1**) are stacked on each other, forming piles of nucleobases and sugar residues as shown in Fig. 4 ▸(*a*). Details of the arrangement of the individual mol­ecules are highlighted in Figs. 4 ▸(*b*) and 4(*c*). As 8-furyl­imidazolo-dC (**1**) shows strong self-pairing properties within the DNA double helix (Jana *et al.*, 2015 ▸), with a head-to-head alignment of the nucleobases in anti­parallel-stranded (aps) DNA and a head-to-tail arrangement in parallel-stranded (ps) DNA, we assumed that one of these two pairing motifs will be observed in the solid-state structure of ^fur^ImidC (**1**). In fact, we found a kind of head-to-tail arrangement of two neighbouring nucleoside mol­ecules, but with a notable tilt of the mol­ecules with respect to each other. As a consequence, instead of two expected N—H⋯N hydrogen bonds with N9 of the imidazole ring of one mol­ecule as hydrogen-bond donor and N3 of the pyrimidine ring of another mol­ecule as acceptor, only one of these contacts is found for neighbouring mol­ecules (N9—H9⋯N3^i^; for hydrogen-bonding data and symmetry codes, see Table 3 ▸). Due to the tilted arrangement of the mol­ecules, the second possible N9—H9⋯N3^i^ contact is observed to a ^fur^ImidC (**1**) mol­ecule of another layer [Fig. 4 ▸(*c*)]. In addition, hydrogen bonding is observed between the nucleobase, with N7 and O2 as acceptors and O3′ (O3′*A*—H3′1*A*⋯N7^ii^) as well as O5′ (O5′*A*—H5′*A*⋯O2^iii^) of the sugar residue as hydrogen-bond donors [Fig. 4 ▸(*a*)]. The furyl substituent does not participate in hydrogen bonding.

### Hirshfeld surface analysis of ^fur^ImidC (1)

**Figure 5:**
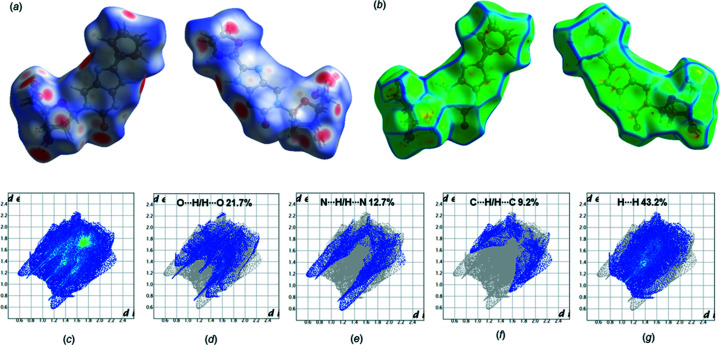
(*a*) Hirshfeld surface of ^fur^ImidC (**1**) mapped with *d*_norm_ (−0.5 to 1.5 Å), shown in front and back view. (*b*) Curvedness surface plots (front and back view; −4.0 to 0.4 Å). 2D fingerprint plots showing the percentage contributions of various inter­actions to the total Hirshfeld surface area of com­pound **1**: (*c*) full inter­actions and resolved contacts; (*d*) O⋯H/H⋯O; (*e*) N⋯H/H⋯N; (*f*) C⋯H/H⋯C; (*g*) H⋯H.

Hirshfeld surface analysis, including 3D surfaces and two-dimensional (2D) fingerprint plots, represent a convenient method for obtaining and visualizing information on inter­molecular inter­actions (Spackman & Jayatilaka, 2009 ▸). The program *CrystalExplorer* (Version 17; Spackman & Jayatilaka, 2009 ▸; Turner *et al.*, 2017 ▸) was used to conduct the Hirshfeld surface analysis of 8-furyl­imidazolo-dC (**1**) mapped with a *d*_norm_ range of −0.5 to 1.5 Å, shape index (−1.0 to 1.0 Å; Fig. S1 in the supporting information) and curvedness (−4.0 to 0.4 Å), as well as the corresponding 2D fingerprint plots. The individual hydrogen-bonding inter­actions were identified on the *d*_norm_ surfaces as large circular areas (intense red spots). These red areas indicate the close N—H⋯N, O—H⋯N and O—H⋯O contacts, as these inter­actions are shorter than the sum of the van der Waals radii and show negative *d*_norm_ [Fig. 5 ▸(*a*)]. The results of the Hirshfeld analyses are consistent with the hydrogen-bonding data (Table 3 ▸). Moreover, the large flat region across the nucleobase of ^fur^ImidC (**1**) visible on the curvedness surface plots indicates π–π inter­actions [Fig. 5 ▸(*b*)]. This observation fits the pronounced stacking inter­actions of mol­ecule **1**, as indicated in Fig. 4 ▸(*a*). Fig. 5 ▸(*c*) shows the overall 2D fingerprint plot of 8-furyl­imidazolo-dC (**1**) and those resolved into O⋯H/H⋯O, N⋯H/H⋯N, C⋯H/H⋯C and H⋯H contacts [Figs. 5 ▸(*d*)–(*g*)], together with their relative contributions to the Hirshfeld surface. The proportions of O⋯H/H⋯O, N⋯H/H⋯N and C⋯H/H⋯C inter­actions com­prise 21.7, 12.7 and 9.2%, respectively, of the total Hirshfeld surfaces.

### ^fur^ImidC solvent-dependent fluorescence

**Figure 6:**
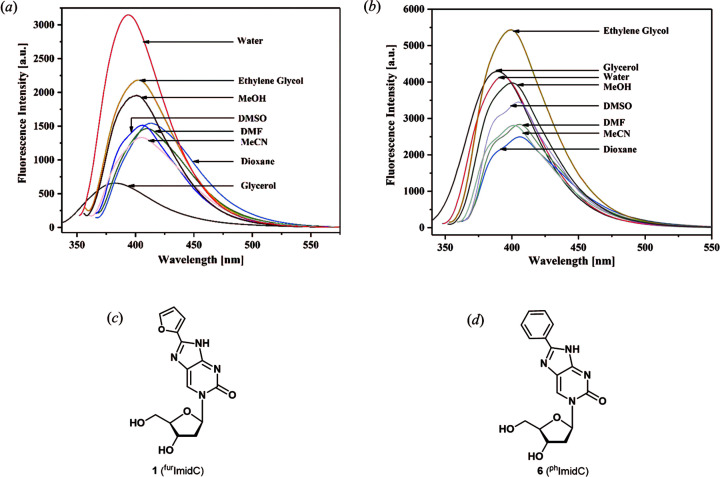
Fluorescence emission spectra measured in solvents of different polarity using a nucleoside concentration of 3 µ*M* and excitation at λ_abs,max_ for (*a*) ^fur^ImidC (**1**) and (*b*) ^ph^ImidC (**6**). The structures of (*c*) 8-furyl­imidazolo-dC (^fur^ImidC, **1**) and (*d*) 8-phenyl­imidazolo-dC (^ph^ImidC, **6**).

The nucleobases of the purine or pyrimidine nucleosides are virtually nonfluorescent. They become fluorescent when a furyl moiety is attached (Greco & Tor, 2007 ▸; Sinkeldam *et al.*, 2011 ▸). On the contrary, the nucleobase of ^fur^ImidC (**1**) shows already intrinsic fluorescence (Seela *et al.*, 1994 ▸, 2000 ▸; Seela & Chen, 1995 ▸). Earlier, it was reported that a furyl substituent attached to a pyrimidine or purine nucleobase *via* a rotatable ar­yl–aryl bond represents a mol­ecular rotor (Lee *et al.*, 2018 ▸). However, it was not discussed that axial chirality is introduced into these mol­ecules and atropisomers are formed. This information can be drawn from the crystal structure of ^fur^ImidC (**1**). Similar to the situation in the crystalline state, the rotation becomes hindered in solvents of high viscosity and therefore a strong impact on the fluorescence is expected. This prompted us to record the fluorescence emission spectra of ^fur^ImidC (**1**) in solvents of different polarity and viscosity [Fig. 6 ▸(*a*)]. For com­parison, the fluorescence spectra of ^ph^ImidC (**6**) were also recorded [Fig. 6 ▸(*b*)]. This mol­ecule carries a less polar phenyl substituent which might show weaker inter­actions with solvent mol­ecules. For Stokes shifts, quantum yields and brightness, see Table S1 in the supporting information.

Already the UV spectra of **1** shows a solvent dependence of the long wavelength maximum centred around 350 nm (Fig. S2 in the supporting information). In the nonpolar solvents DMSO and di­methyl­formamide (DMF), the UV maxima are bathochromically shifted by around 10 nm com­pared to the maxima recorded in the polar solvents glycerol and water. For com­parison, the UV spectra determined for ^ph^ImidC (**6**) show similarities but also differences with respect to **1** (Fig. S2 in the supporting information). Herein the maxima at shorter wavelengths are hypsochromically shifted by around 10 nm.

Much stronger solvent dependencies are observed for the fluorescence spectra. Excitation was carried out at the long wavelength maximum of each solvent (Table S1 in the supporting information). The fluorescence of ^fur^ImidC (**1**) depends strongly on the particular solvent [Fig. 6 ▸(*a*)] and much higher quantum yields are observed in water (Φ = 0.69) com­pared to all other solvents (Table S1). The fluorescence intensity in aprotic solvents of low polarity (DMSO, DMF, MeCN and dioxane) is lower. For nucleoside **6**, the situation is different. The fluorescence intensity is highest in polar protic solvents [Fig. 6 ▸(*b*)]. However, the highest quantum yield for **6** was observed in ethyl­ene glycol (Φ = 0.65; Table S1).

The fluorescence obtained in glycerol [*E*_T_(30) = 57.0 kcal mol^−1^, η = 1.412  Pa  s] and ethyl­ene glycol [*E*_T_(30) = 56.3 kcal mol^−1^, η = 1.61 × 10 ^−2^ Pa s] are of particular inter­­est, as both solvents exhibit almost com­parable polarity but different viscosity (Reichardt, 1994 ▸). Indeed, the fluorescence intensity of ^fur^ImidC (**1**) responds significantly to the viscosity difference. In glycerol, the fluorescence is the weakest (Φ = 0.22), while it is much higher in ethyl­ene glycol (Φ = 0.46) (Table S1). Most inter­estingly, ^fur^ImidC (**1**) shows low fluorescence in the highly viscous solvent glycerol and strong fluorescence in the lowly viscous solvent water. For the phenyl-substituted nucleoside **6**, the effect is much weaker. Accordingly, ^fur^ImidC (**1**) responds strongly to microenvironmental changes (polarity and viscosity) and is in particular sensitive against viscosity changes. In fact, it has to be considered that in solution already two atropisomeric mol­ecules are present. This is not only valid for com­pound **1**, but is a general phenomenon occurring in related purine and pyrimidine nucleosides with unsymmetric heterocycles as side chains, as reported by Tor (Greco & Tor, 2007 ▸) and others (Tokugawa *et al.*, 2016 ▸).

## Conclusion

The crystal structure of fluorescent 8-furyl­imidazolo-2′-de­oxy­cytidine (^fur^ImidC, **1**) has been studied. In the solid state, ^fur^ImidC shows two independent types of disordered groups (sugar and furyl moiety). The sugar residue shows a minor disorder (87:13 split), while the disorder of the furyl ring shows a split of ∼30:70. The latter results from atropisomerism at the C8—C2′′ chiral axis connecting the nucleobase and the furyl residue. The isomers are related to each other by a 180° rotation, are coplanar and are therefore in conjugation.

For the solid-state conformational analysis of ^fur^ImidC (**1**), only atoms with the predominant occupancy were used. Nucleoside **1** shows an *anti* conformation at the glycosylic bond [χ = −147.2 (7)°] and a C2′-*endo* (*S*, ^2^ *T*_1_) sugar pucker. In solution, the *S* conformation also predominates, as shown by a ^1^H NMR-based conformational analysis of the furan­ose puckering. Stacking inter­actions of the mol­ecules, as well as hydrogen bonding between two nucleobase moieties and between the nucleobase and the sugar residue, stabilize the crystal structure. Two neighbouring ^fur^ImidC mol­ecules are arranged in a head-to-tail fashion, but with a notable tilt of the mol­ecules with respect to each other. Consequently, one N9—H9⋯N3 hydrogen bond is found for neighbouring mol­ecules within one layer, while a second contact with N9 as hydrogen-bond donor and N3 as acceptor is formed to a mol­ecule of an adjacent layer.

The ^fur^ImidC (**1**) nucleoside shows fluorescence. The fluorescence intensity responds strongly to the viscosity of the solvent due to atropisomerism and the conjugation of the furan system with the imidazolo–pyrimidine heterocycle. This environmental phenomenon might be used to monitor conformational changes in nucleic acids or inter­actions with proteins.

## Supplementary Material

Crystal structure: contains datablock(s) I, global. DOI: 10.1107/S2053229622001000/cu3177sup1.cif

Structure factors: contains datablock(s) I. DOI: 10.1107/S2053229622001000/cu3177Isup2.hkl

UV spectra, photophysical data, 1H NMR chemical shifts and 1H NMR spectrum. DOI: 10.1107/S2053229622001000/cu3177sup3.pdf

CCDC reference: 2095197

## Figures and Tables

**Table 1:** Experimental details

Crystal data	
Chemical formula	C_14_H_14_N_4_O_5_
*M*_r_	318.29
Crystal system, space group	Orthorhombic, *P*2_1_2_1_2_1_
Temperature (K)	100
*a*, *b*, *c* (Å)	5.3043 (4), 11.1405 (8), 22.6067 (18)
*V* (Å^3^)	1335.89 (18)
*Z*	4
Radiation type	Cu *K*α
μ (mm^−1^)	1.04
Crystal size (mm)	0.38 × 0.06 × 0.03

Data collection	
Diffractometer	Bruker D8 Venture PHOTON 100 CMOS
Absorption correction	Multi-scan (*SADABS*; Bruker, 2014 ▸)
*T*_min_, *T*_max_	0.69, 0.97
No. of measured, independent and observed [*I* > 2σ(*I*)] reflections	28381, 2241, 1559
*R*_int_	0.149
(sin θ/λ)_max_ (Å^−1^)	0.588

Refinement	
*R*[*F* ^2^ > 2σ(*F* ^2^)], *wR*(*F* ^2^), *S*	0.053, 0.142, 1.03
No. of reflections	2241
No. of parameters	335
No. of restraints	279
H-atom treatment	H atoms treated by a mixture of independent and constrained refinement
Δρ_max_, Δρ_min_ (e Å^−3^)	0.22, −0.20
Absolute structure	Flack *x* determined using 503 quotients [(*I* ^+^) − (*I* ^−^)]/[(*I* ^+^) + (*I* ^−^)] (Parsons *et al.*, 2013 ▸)
Absolute structure parameter	0.0 (3)

Computer programs: *APEX3* (Bruker, 2016 ▸), *SAINT* (Bruker, 2015 ▸), *SHELXT2014* (Sheldrick, 2015*a* ▸), *SHELXL2018* (Sheldrick, 2015*b* ▸), *shelXle* (Hübschle *et al.*, 2011 ▸) and *SHELXL2014* (Sheldrick, 2015*b* ▸).

**Table 2:** Selected geometric parameters (Å, °)

O2—C2	1.244 (6)	N1—C1′*A*	1.47 (3)
C8—C2′′*A*	1.418 (19)	N1—C1′	1.477 (8)
C8—C2′′	1.451 (11)		
			
N7—C8—C2′′*A*	116.1 (14)	C2—N1—C1′*A*	117 (2)
N9—C8—C2′′*A*	130.4 (14)	C2—N1—C1′	117.4 (6)
N7—C8—C2′′	128.2 (8)	O5′—C5′—C4′	110.8 (8)
N9—C8—C2′′	118.3 (8)	O5′*A*—C5′*A*—C4′*A*	114 (4)
			
N9—C8—C2′′—O1′′	177.7 (12)	C3′—C4′—C5′—O5′	47.3 (10)
N9—C8—C2′′*A*—O1′′*A*	1 (5)	C2—N1—C1′*A*—O4′*A*	−162 (3)
C2—N1—C1′—O4′	−147.2 (7)	C3′*A*—C4′*A*—C5′*A*—O5′*A*	164 (6)

**Table 3:** Hydrogen-bond geometry (Å, °)

*D*—H⋯*A*	*D*—H	H⋯*A*	*D*⋯*A*	*D*—H⋯*A*
N9—H9⋯N3^i^	0.87 (3)	2.01 (3)	2.863 (7)	165 (6)
O3′*A*—H3′1*A*⋯N7^ii^	0.84	2.08	2.852 (16)	153
O5′*A*—H5′*A*⋯O2^iii^	0.84	1.99	2.818 (6)	169

Symmetry codes: (i) 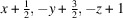 ; (ii) 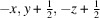 ; (iii) 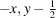 , 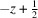 .
