# Supplementary material for: 8-Furyl­imidazolo-2′-de­oxy­cytidine: crystal structure, packing, atropisomerism and fluorescence
Source: Acta Crystallogr C Struct Chem. 2022 Feb 9;78(Pt 3):141–7. doi: 10.1107/S2053229622001000 (PMC8896525; doi:10.1107/S2053229622001000)
Supplement: Supplementary file 3 [file c-78-00141-sup3.pdf]

STRUCTURAL CHEMISTRY

Volume 78 (2022)

Supporting information for article:

8-Furylimidazolo-2′-deoxycytidine: crystal structure, packing, atropisomerism and fluorescence

Simone Budow-Busse, Sunit K. Jana, Dasharath Kondhare, Constantin Daniliuc and Frank Seela

Table of Contents

Figure S1. Shape index of ^{fur}ImidC 3

Figure S2. UV spectra measured in solvents of different polarity and viscosity 3

Fluorescence studies 4

Table S1. Photophysical data of **1** and **6** in solvents of different polarities 4

Table S2. ^{1}H NMR chemical shifts, proton-proton vicinal and geminal coupling constants and conformation of the nucleoside sugar residue of ^{fur}ImidC (**1**) 5

Literature 5

Figure S3. Sugar conformation of nucleosides 5

Figure S4. ^{1}H NMR spectrum of ^{fur}ImidC 6

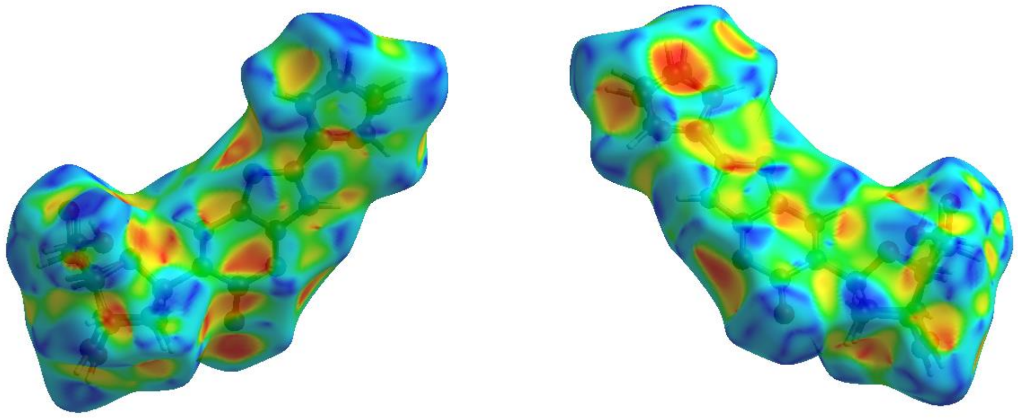

(a) (b)

Figure S1. Shape index (-1.0 to 1.0 Å) of ^{fur}ImidC (**1**). (*a*) Front view. (*b*) Back view.

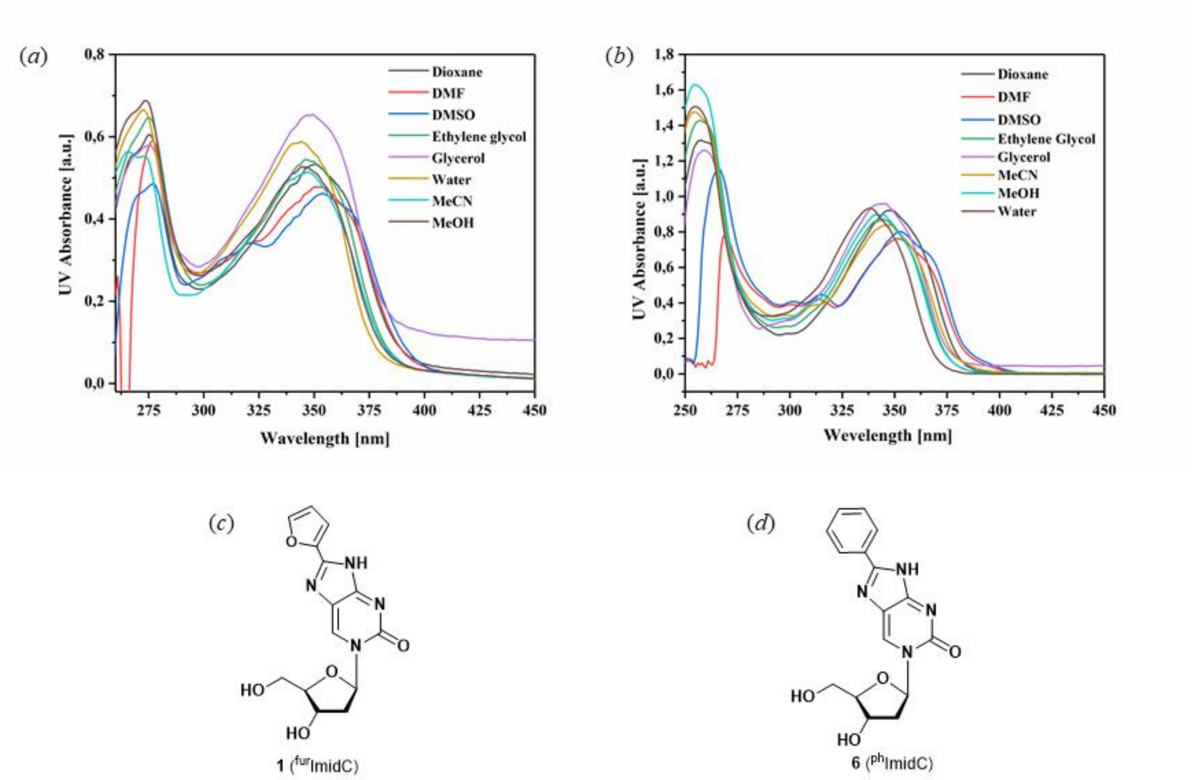

Figure S2. UV spectra measured in solvents of different polarity and viscosity using a nucleoside concentration of 3 µ*M*. (*a*) ^{fur}ImidC (**1**). (*b*) ^{ph}ImidC (**6**). (*c*) Structure of 8-furyl-imidazolo-dC (^{fur}ImidC, **1**). (*d*) Structure of 8-phenyl-imidazolo-dC (^{ph}ImidC, **6**).

Fluorescence Studies

Fluorescence measurements were performed with a F-2500 fluorescence spectrophotometer. Fluorescence spectra of nucleosides **1** and **6** were measured in different solvents and were performed with identical concentrations of 3 µ*M*. Quantum yields Φ were determined by using quinine sulfate in 0.1 M sulfuric acid as a standard with $\Phi = 0.54$. The quantum yield Φ (x) of the compound (**1** or **6**) can be calculated by the following equation:

$$\Phi\,(x) = \Phi\,(ST)(AST/AX)(FX/FST)(\eta^2X/\eta^2ST)$$

where Φ (ST) is the quantum yield of the standard, A is the absorbance at the excitation wavelength, F is the integrated area of the emission curve, the subscripts X and ST refer to unknown and standard and η is the refractive index of solvent.

Table S1. Photophysical data of imidazole-dC nucleosides **1** and **6** measured in solvents of different polarities.[a]

Compound	Solvent	$\lambda_{abs, max}$ Ex [nm]	$\lambda_{max, em}$ Em [nm]	Stokes shift (Δν)[b] [cm^{-1}]	Φ[c]	ε_{max} [mol^{-1}dm^{3}cm^{-1}]	Brightness[d]
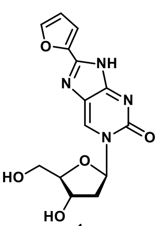 1	Water	342	394	3900	0.69	8400	5800
	Ethylene glycol	347	403	4000	0.46	7800	3600
	MeOH	346	401	4000	0.48	7500	3600
	DMF	356	410	3700	0.48	6700	3200
	Dioxane	356	413	3900	0.49	7300	3600
	MeCN	350	405	3900	0.39	7200	2800
	DMSO	356	406	3500	0.48	6500	3100
	Glycerol	319	383	5200	0.22	6000	1300
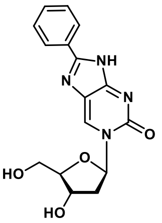 6 (^{ph}ImidC)	Water	338	394	4200	0.55	13400	7300
	Ethylene glycol	343	400	4200	0.65	12400	8000
	MeOH	342	400	4200	0.56	12800	7200
	DMF	353	406	3700	0.52	10900	5700
	Dioxane	351	406	3900	0.41	12900	5300
	MeCN	349	402	3700	0.43	11900	5100
	DMSO	354	405	3600	0.58	11400	6600
	Glycerol	313	390	6300	n.a.[e]	10500	7500

[a] The concentration of nucleosides was 3 µ*M*. [b] The Stokes shift was calculated from the equation $\Delta E_{photon} = hc(1/\lambda_{abs,max} - 1/\lambda_{max, em})$. [c] The fluorescence quantum yields (Φ) were calculated using quinine sulfate (3 µ*M*) in 0.1 M H_2SO_4 ($\Phi_{St} = 0.54$). [d] Brightness was calculated as: brightness = $\varepsilon_{max}\,\Phi$. [e] n.a. corresponds to not available.

Table S2. ^{1}H NMR chemical shifts, proton-proton vicinal and geminal coupling constants and conformation of the nucleoside sugar residue of ^{fur}ImidC (**1**) in solution.[a,b]

	Chemical shift/ppm										
	H-1’	H-2’	H-2”	H-3’	H-4’	H-5’	H-5”	3’-OH	5’-OH	NH	H6
**1**	6.22 (t)	2.09 (dt)	2.37 (ddd)	4.26 (q)	3.91 (q)	3.64 (dt)	3.73 (dt)	5.26 (m)	5.26 (m)	13.11 (s)	8.90 (s)
	Coupling constant/Hz [*J*(H,H)]									Conformation	
	1’2’	1’2”	2’2”	2’3’	2”3’	3’4’	4’5’	4’5”	5’5”	%*N*	%*S*
**1**	6.0	6.1	-13.2	5.7	4.5	4.1	4.3	3.9	-12.3	43	57

[a] Measured in DMSO-d_6 at 298 K; rms < 0.4 Hz. H-2’= H-2′$_{\beta}$; H-2’’= H-2′$_{\alpha}$. [b] For *PSEUROT* (Van Wijk *et al.*, 1999) calculations, the coupling constants ^{3}J(H1’-H2’), ^{3}J(H1’-H2”), ^{3}J(H2’-H3’), ^{3}J(H2”-H3’) and ^{3}J(H3’-H4’) were used.

Literature

Van Wijk, L., Haasnoot, C. A. G., de Leeuw, F. A. A. M., Huckriede, B. D., Westra Hoekzema, A. J. A. & Altona, C. (1999). *PSEUROT 6.3*. Leiden Institute of Chemistry, Leiden University, The Netherlands.

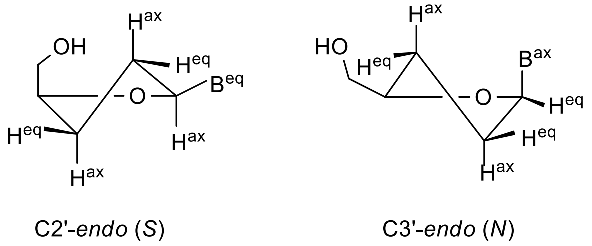

Figure S3. Sugar conformation of nucleosides. B corresponds to nucleobase. ax: axial; eq: equatorial.

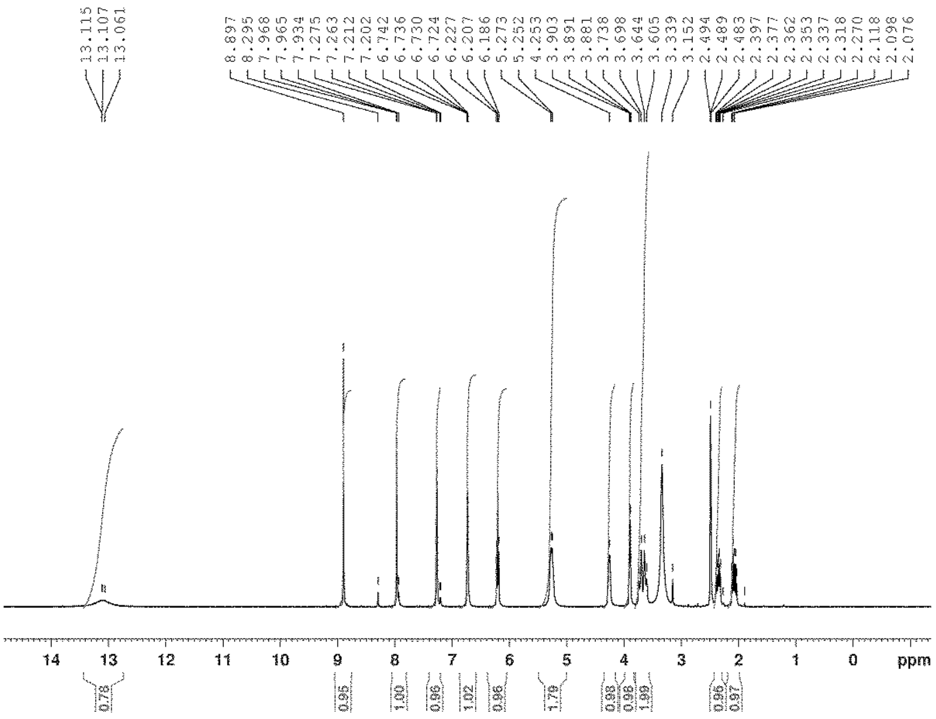

Figure S4. ^{1}H NMR (300 MHz, DMSO-d_6) spectrum of ^{fur}ImidC (**1**).
